# Emergency Surgery in the elderly patient

**DOI:** 10.1186/1471-2318-11-S1-A30

**Published:** 2011-08-24

**Authors:** Antonio Martino, Ciro De Martino, Gautam Maharajan, Marco Evangelista, Rosa Maria Giamattei, Anna Pisapia

**Affiliations:** 1 Casa di Cura “A. Grimaldi” di San Giorgio a Cremano (NA). Dipartimento di Chirurgia, Italy; 2 Università degli Studi di Napoli “Federico II”. U.O.C. di Chirurgia Generale, Italy

## Background

Advances in surgical and anesthetic techniques combined with sophisticated perioperative monitoring are factors that have contributed to an expanding number of older adults undergoing surgery. Older persons often have multiple comorbid conditions that limit their functional capacity and increase the risk of death. An initial complication is much more likely to lead to other complications; failure of one organ, leads to failure of other organs.

## Methods

A preoperative assessment is useful to identify factors associated with increased risks of specific complications and to recommend a management plan that minimizes the risks. Each person should be assessed individually, and judgments should be based on an individual's problem and physiologic status, not on age alone.

## Results

Advanced age, poor functional status at baseline, impaired cognition, and limited support at home are risk factors for adverse outcomes. However, when age and severity of illness are directly compared, severity of illness is a much better predictor of outcome compared to age. Emergency operations carry a greater risk compared to elective operations in all age groups, particularly elderly persons.

## Conclusions

The ageing process of general population implies new socio-sanitary problems. Indications for surgical intervention have been modified and enhanced. As far as elective surgery is concerned, the results in elderly subjects do not seem alarming, whereas less satisfactory results have been registered in the patients who underwent emergency surgery, where nowadays morbidity and mortality are still high.

It will be possible to obtain better results through geriatric surgery only by reducing emergency interventions as much as possible. In order to do so, it is important to insist on intervening before the illness, during its natural evolution which requires actions that cannot be postponed. This would lead to positive results not only in terms of mortality and morbidity, which are still considered the main targets, but also in terms of length of hospital stay and rehabilitation.
